# Accidental Hydrogen Peroxide Ingestion

**DOI:** 10.5811/cpcem.2018.4.37749

**Published:** 2018-05-18

**Authors:** Rohit B. Sangal, Jonathan Bar, Brian Weiss, Matthew Kelly, Timothy Medina

**Affiliations:** * Perelman School of Medicine at the University of Pennsylvania, Department of Emergency Medicine, Philadelphia, Pennsylvania; † Perelman School of Medicine at the University of Pennsylvania, Department of Emergency Medicine, Division of Undersea and Hyperbaric Medicine, Philadelphia, Pennsylvania; ‡ Lancaster General Hospital, Department of Emergency Medicine, Lancaster, Pennsylvania

## CASE PRESENTATION

**Image 1:**
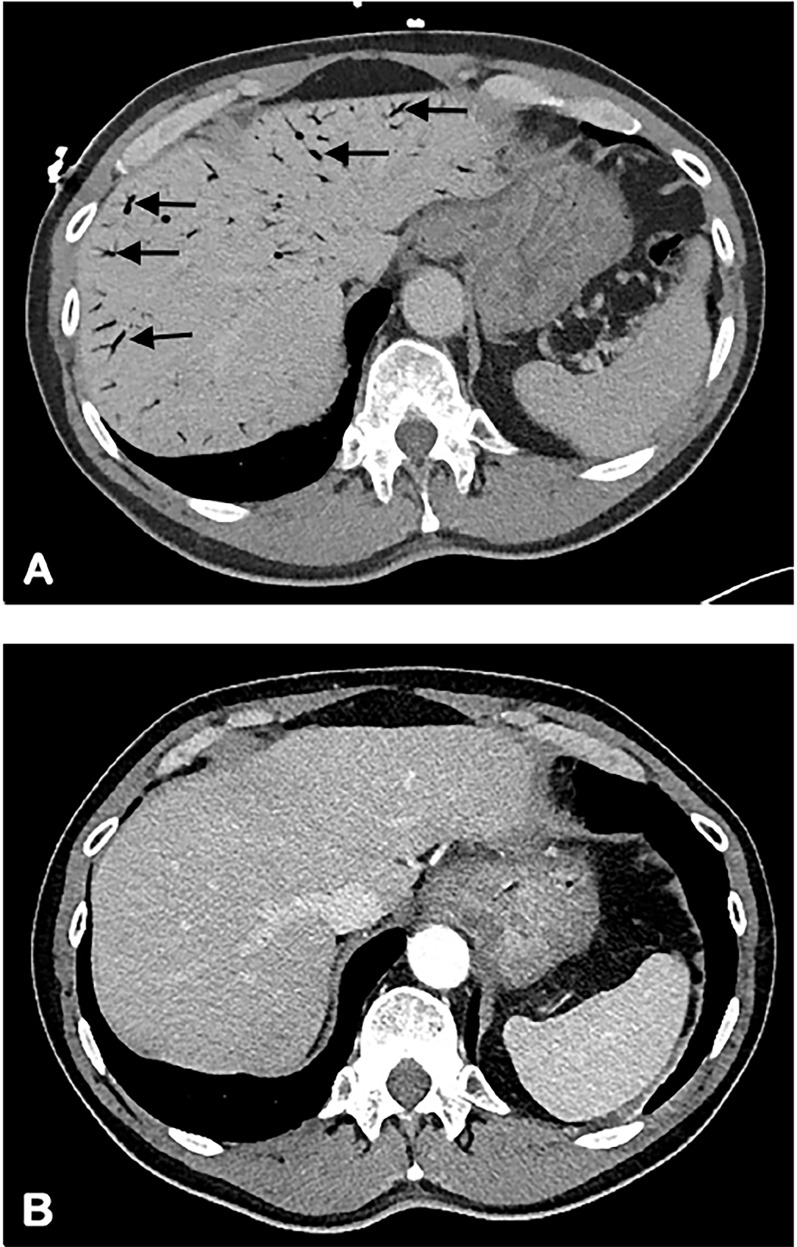
Computed tomography of abdomen showing portal venous gas of the liver before hyperbaric oxygen therapy (arrows) (A) and resolution after therapy (B).

A 69-year-old male with no significant past medical history presented to the emergency department (ED) after accidental ingestion of hydrogen peroxide. He used concentrated hydrogen peroxide as a home remedy. Intending to drink water, he had accidentally grabbed the incorrect bottle and ingested “multiple gulps.” He soon started to experience multiple symptoms including eructation, flatulence, nausea, non-bloody vomiting and generalized abdominal pain. His computed tomography is shown (Image 1A). During his stay in the ED he started to complain of headache, blurry vision and was found to have a left homonymous hemianopia, dysmetria and hyperreflexia. He was emergently transferred to a tertiary care hospital for hyperbaric therapy.

## DISCUSSION

The patient was diagnosed with portal venous gas and presumed cerebral air embolism. Concentrated hydrogen peroxide (>10%) is primarily an industrial chemical (as opposed to the 3% concentration sold for consumer use), but it is also used as a natural remedy when diluted for “hyperoxygen” therapy.^1^,^2^ These ingestions not only cause direct caustic injury but the resulting exothermic reaction liberates large volumes of oxygen that distend the stomach and, if not expelled, diffuse into the blood stream and tissues.^3^ Some studies report that hyperbaric therapy improves outcomes from cerebral infarction secondary to air embolism.^1^,^2^

Our patient, already experiencing a visual field deficit, emergently received hyperbaric therapy over advanced head imaging given concern for neurologic sequelae. His follow-up abdominal computed tomography less than 24 hours after completing hyperbaric therapy showed complete resolution of portal venous gas (Image 1B). His gastrointestinal and neurologic symptoms resolved, and he returned to baseline.

Documented patient informed consent and/or Institutional Review Board approval has been obtained and filed for publication of this case report.

**CPC-EM Capsule**

What do we already know about this clinical entity?

Hydrogen peroxide is a common household item that has a variety of uses including as a home remedy. As such, providers need to be aware of the effects of toxic ingestions.

What is the major impact of the image(s)?

While portal venous gas can develop quickly and be extensive, it can be reversible with quick recognition and treatment.

How might this improve emergency medicine practice?

Providers should have a heightened awareness of the effect of this household ingestion and have a low threshold to image or observe patients for progression of symptoms.
